# *Aeromonas hydrophila* inhibits autophagy triggering cytosolic translocation of mtDNA which activates the pro-apoptotic caspase-1/IL-1β-nitric oxide axis in headkidney macrophages

**DOI:** 10.1080/21505594.2021.2018767

**Published:** 2021-12-30

**Authors:** Manmohan Kumar, Asha Shelly, Priyanka Dahiya, Atish Ray, Shibnath Mazumder

**Affiliations:** a Immunobiology Laboratory, Department of Zoology, University of Delhi, Delhi, India; b Faculty of Life Sciences and Biotechnology, South Asian University, New Delhi, India

**Keywords:** *Aeromonas hydrophila*, head kidney macrophage, er-stress, mtROS, mtDNA, autophagy, apoptosis, caspase-1, IL-1β

## Abstract

The molecular mechanisms underlying *Aeromonas hydrophila*-pathogenesis are not well understood. Using head kidney macrophages (HKM) of *Clarias gariepinus*, we previously reported the role of ER-stress in *A. hydrophila*-induced pathogenesis. Here, we report that PI3K/PLC-induced cytosolic-Ca^2+^ imbalance induces the expression of pro-apoptotic ER-stress marker, CHOP in *A. hydrophila-*infected HKM. CHOP promotes HKM apoptosis by inhibiting AKT activation and enhancing JNK signaling. Elevated mitochondrial ROS (mtROS) was recorded which declined significantly by ameliorating ER-stress and in the presence of ER-Ca^2+^ release modulators (2-APB and dantrolene) and mitochondrial-Ca^2+^ uptake inhibitor, Ru360, together suggesting the role of ER-mitochondrial Ca^2+^ dynamics in mtROS generation. Inhibiting mtROS production reduced HKM death implicating the pro-apoptotic role of mtROS in *A. hydrophila*-pathogenesis. The expression of autophagic proteins (LC3B, beclin-1, and atg 5) was suppressed in the infected HKM. Our results with autophagy-inducer rapamycin demonstrated that impaired autophagy favored the cytosolic accumulation of mitochondrial DNA (mtDNA) and the process depended on mtROS levels. Enhanced caspase-1 activity and IL-1β production was detected and transfection studies coupled with pharmacological inhibitors implicated mtROS/mtDNA axis to be crucial for activating the caspase-1/IL-1β cascade in infected HKM. RNAi studies further suggested the involvement of IL-1β in generating pro-apoptotic NO in *A. hydrophila*-infected HKM. Our study suggests a novel role of ER-mitochondria cross-talk in regulating *A. hydrophila* pathogenesis. Based on our observations, we conclude that *A. hydrophila* induces ER-stress and inhibits mitophagy resulting in mitochondrial dysfunction which leads to mtROS production and translocation of mtDNA into cytosol triggering the activation of caspase-1/IL-1β-mediated NO production, culminating in HKM apoptosis.

## Introduction

*A. hydrophila*, a Gram-negative bacterium is capable of infecting a wide range of organisms. In fish, it is known to cause ulcerative disease syndrome (UDS) while in humans it causes serious health complications [1]. Although, the mechanism underlying *A. hydrophila* pathogenesis is not well understood; at the cellular level, the major outcome of this host-pathogen interaction is apoptosis of the host cells [2,3].

Pathogen-induced alterations in cytosolic Ca^2+^ [(Ca^2+^)_C_] levels have been found to be crucial for apoptosis of the host macrophages [4]. ER is the chief location for synthesis and correct folding of cellular proteins which is highly dependent on (Ca^2+^)_C_ homeostasis [5]. Perturbation in (Ca^2+^)_C_ homeostasis interferes with normal ER protein load and functioning of the organelle, leading to stress condition termed as ER-stress [6,7]. To overcome ER-stress, eukaryotic cells have evolved the unfolded protein response (UPR), characterized by induction of IRE1 driven BiP and PERK-eIF2 mediated expression of CHOP [6,8]. ER-stress is essential for the survival of cells; however, prolonged ER-stress can instigate apoptosis [5,6,9,10]. CHOP is considered a well-known marker for ER-stress induced apoptosis [11].

Among the different intracellular signaling molecules that trigger (Ca^2+^)_C_ release, PI3K/PLC is important [12]. Different bacterial ligands have been reported to activate PI3K/PLC mediated release of Ca^2+^ from intracellular sources thus modulating several downstream signaling molecules [13]. The pro-apoptotic role of PI3K-PLC-mediated (Ca^2+^)_C_ alteration has recently been established in *A. hydrophila*-pathogenesis [14]. Though, *A. hydrophila*-induced ER-stress has been testified earlier [3]; the involvement of PI3K-PLC axis in initiating ER-stress induced apoptosis has not been reported in *A. hydrophila*-pathogenesis.

In order to overcome stress, ER releases Ca^2+^ [(Ca^2+^)_ER_] *via* ER membrane receptors [15]. The (Ca^2+^)_ER_ is propelled out through specific channels [16] and enters into the mitochondria *via* mitochondrial calcium uniporters (MCU) and voltage-dependent anion channels (VDAC), and the process is aided by the juxtaposition of the two organelles [17–19]. The mechanism of ER-stress-induced apoptosis is not well known, but there are reports implicating the cross-talk between ER and mitochondria are critical in different model systems including fish [3,5].

Several earlier reports have documented a correlation between innate immune signaling and mitochondrial functioning [20,21]. (Ca^2+^)_ER_ on entering the mitochondria impairs the ETC causing excessive ROS production by the mitochondria [22,23]. There are few reports suggesting mitochondrial ROS (mtROS) as one of the major contributory factors in host cell bactericidal activity, although the exact mechanism remains obscure [21]. Additionally, the role of mtROS in microbial pathogenesis is not well elucidated in fish.

Recent reports suggest that mitochondrial DNA (mtDNA) acts as a modulator of immune responses [24,25]. Under varied conditions of stress, mtDNA is translocated into the cytosol and is sensed as a damage-associated molecular pattern (DAMP) by immune receptors triggering downstream signaling cascade to produce immune-effector molecules [25]. The involvement of mtDNA in regulating host immunity to microbial immunity is fairly well established [24,26]. It has also been observed that mtROS plays an important role in triggering the release of mtDNA in cytoplasm impacting host immune responses [21,27,28]. Autophagy is a conserved cellular quality control mechanism which facilitates the selective turnover of damaged cell-organelles, including mitochondria [29]. Suppression of autophagy machinery led to cytosolic translocation of mtDNA in macrophages [27]. To the best of our knowledge, the role of mtROS/mtDNA axis in regulating microbial pathogenesis has not been reported in fish.

One of the major outcomes of ER-mitochondrial interaction is inflammasome formation, leading to caspase-1 activation [30]. Though there is very little information on inflammasomes in fish, the presence of signature molecules like caspase-1 and IL-1β in several fish [31–34] suggests similar mechanism helps them to respond to varying insults, like higher vertebrates [35]. Caspase-1 has been reported to be conserved from teleosts to mammals in terms of specificity, processing, and function [33]. During caspase-1 activation, pro-caspase-1 is processed into active caspase-1 [30], but the existence of alternatively spliced isoforms of caspase-1 has also been reported in fish [33]. There are several reports documenting the activation of caspase-1 in response to *A. hydrophila* [34].

The pro-inflammatory cytokine IL-1β is produced by innate immune cells in response to viral [36], bacterial [37], and parasitic infection [38]. Although IL-1β has been reported in fishes [39–43], but the synthesis of mature IL-1β is debatable. There are reports suggesting IL-1β processing to be both caspase-1 dependent [32,33] and caspase-1 independent in fish [34,44].

Head kidney macrophages (HKMs) from *Clarias gariepinus* have been established as an alternate model to understand *A. hydrophila* pathogenesis at the cellular level [3,14]. *A. hydrophila* is reported to induce activation of an intricate network of signaling molecules ultimately leading to caspase-3-mediated HKM apoptosis [3]. The role of both caspase-9 as well as caspase-8 has been observed in triggering *A. hydrophila-*induced HKM apoptosis [3]. Pro-apoptotic role of (Ca^2+^)_C_ in ER-stress mediated HKM apoptosis is evident in the *A. hydrophila*-pathogenesis [3], the upstream events that initiate the process have not been reported. In the present study, we describe the primal role of PI3K-PLC axis on ER-stress and mtROS generation that triggers the mtDNA/caspase-1/IL-1β axis induced apoptosis of *A. hydrophila*-infected HKM.

## Results

### PI3K-PLC signaling is imperative in triggering *A. hydrophila*-induced ER stress

**Figure 1.**
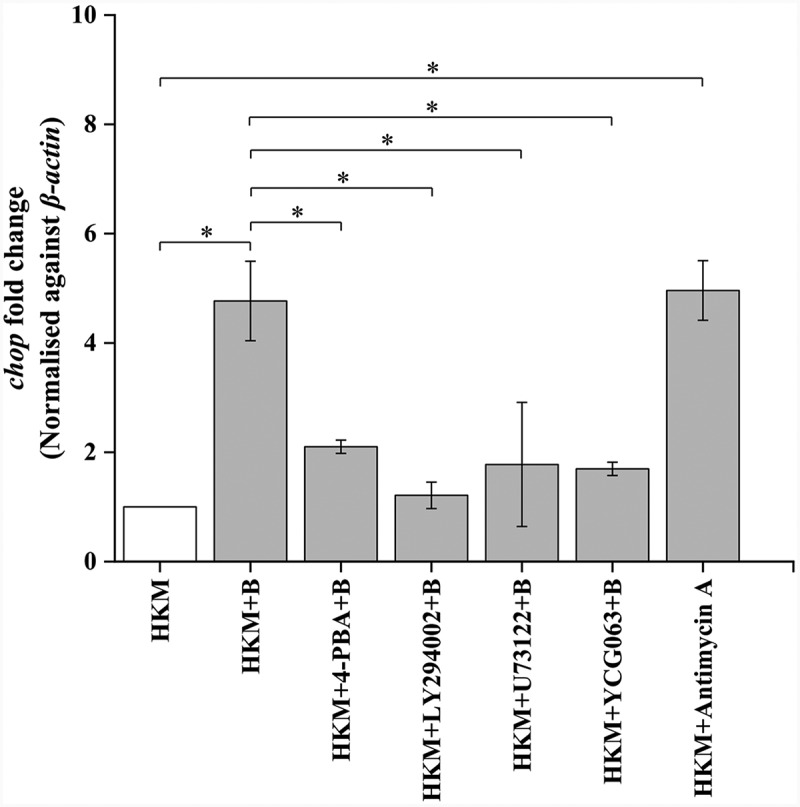
***A. hydrophila*-induced PI3K/PLC axis activates ER-stress**. HKM pre-incubated with 4-PBA, LY-294002, U73122, YCG063, and Antimycin A were infected with or without *A. hydrophila* and *chop* expression studied at 2 h p.i. Vertical bars represent mean ± SE (n = 3). Asterisk indicates significant difference between indicated groups (*p < 0.05). HKM, uninfected HKM; HKM+B, HKM infected with *A. hydrophila*; HKM+4-PBA+B, HKM pre-incubated with 4-PBA infected with *A. hydrophila*; HKM+LY-294002 + B, HKM pre-incubated with LY-294002 infected with *A. hydrophila*; HKM+U73122 + B, HKM pre-incubated with U73122 infected with *A. hydrophila*; HKM+YCG063 + B, HKM pre-incubated with YCG063 infected with *A. hydrophila*, HKM+Antimycin A, HKM pre-incubated with Antimycin A.

We had previously observed the role of ER-stress [3] and PI3K-PLC induced (Ca^2+^)_C_ surge in *A. hydrophila-*induced HKM apoptosis [14]. Hence, our first step was to correlate the two molecular events in *A. hydrophila*-pathogenesis. HKMs were pre-incubated separately with PI3K specific inhibitor, LY294002 and PLC specific inhibitor, U73122, respectively, and *chop* expression monitored by RT-qPCR at 2 h p.i. We had observed *chop* expression to be the maximum at 2 h p.i. in *A. hydrophila-*infected HKM and selected this time point for the study (data not shown). We noted that pre-incubation with LY294002 and U73122 led to significant inhibition of *chop*-mRNA expression (Figure 1). ER-stress inhibitor, 4-PBA was used as control for the study which effectively repressed *chop* mRNA (Figure 1) and protein expression in *A. hydrophila*-infected HKM (Fig. S1). We conclude that PI3K-PLC induced (Ca^2+^)_C_ surge has a primal role in *A. hydrophila*-induced ER stress.

### ER-stress promotes HKM apoptosis by regulating the AKT-JNK signaling cascade

**Figure 2.**
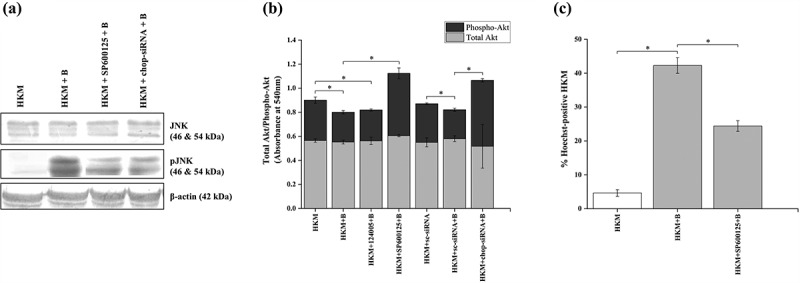
***A. hydrophila*-induced ER-stress induces HKM apoptosis *via* JNK-Akt signaling pathway**. HKM pre-incubated with SP600125, 124,005 or transfected with sc-siRNA or *chop*-siRNA were infected with *A. hydrophila* and at 24 h p.i. (a) phosphorylation of JNK, (b) levels of total and phospho-Akt, and (c) HKM apoptosis was studied. Vertical bars represent mean ± SE (n = 3). Asterisk indicates significant difference between indicated groups (*p < 0.05). HKM, uninfected HKM; HKM+B, HKM infected with *A. hydrophila*; HKM+SP600125 + B, HKM pre-incubated with SP600125 infected with *A. hydrophila*; HKM+124005 + B, HKM pre-incubated with 124,005 infected with *A. hydrophila*; HKM+sc-siRNA, HKM transfected with sc-siRNA (scrambled siRNA); HKM+*chop*-siRNA+B, HKM transfected with *chop*-siRNA infected with *A. hydrophila.*

Pathogen-induced activation of MAPK pathway has been reported in various cellular processes including apoptosis of host macrophages [45]. Toward this direction, we studied the involvement of MAPK family member JNK, which is known to induce apoptosis in several model systems [46]. We measured the levels of total and phosphorylated JNK, respectively, by immunoblotting from cell lysates of infected HKM collected at 24 h p.i. We could detect increased phosphorylation of JNK in the infected HKM (Figure 2a).

We tried to investigate whether ER-stress was possibly activating JNK. The HKMs transfected with *chop*-siRNA were infected and the changes in total and phosphorylated JNK levels studied at 24 h p.i. We observed that *chop*-siRNA effectively inhibited the phosphorylation of JNK in the infected HKM, though the total JNK levels remained unaltered (Figure 2a). The specific pharmacological inhibitor for JNK, SP600125 served as negative control in the study (Figure 2a). This reflects that ER-stress activates JNK in the *A. hydrophila*-infected HKM.

The role of PI3K in HKM apoptosis made us interested to study the role of PI3K-dependent kinase, Akt in the cascade of events. HKMs were transfected with *chop*-siRNA and then infected with *A. hydrophila* and the changes in total and phospho-Akt levels studied using specific ELISA kits. We observed significant reduction in active or phospho-Akt levels in the infected HKM (Figure 2b), though the total Akt levels remained unaltered (Figure 2b). HKM pre-incubated with Akt inhibitor, 124,005 served as positive control for the experiment.

Once we confirmed JNK activation in infected HKM, we set out to determine its consequences. HKM were pre-incubated with SP600125 prior to infection and apoptotic cell death was studied at 24 h p.i. It is evident from Figure 2c that inhibition of JNK significantly inhibited *A. hydrophila-*induced HKM apoptosis. We extended our observation in which we pre-incubated the HKM with SP600125 and studied the changes in total and phospho-Akt consequent to *A. hydrophila* infection. We observed that inhibiting JNK activity resulted in significant increase in phospho-Akt levels (Figure 2b) and inhibited HKM apoptosis (Figure 2c). Pre-incubation with SP600125 had no effect on total Akt levels.

Collectively, these observations suggest (i) JNK activation is downstream event of ER-stress and plays a critical role in HKM apoptosis and (ii) JNK exerts its pro-apoptotic effect by inhibiting Akt activation.

### ER-stress mtROS crosstalk is critical in *A. hydrophila*-induced HKM apoptosis

**Figure 3.**
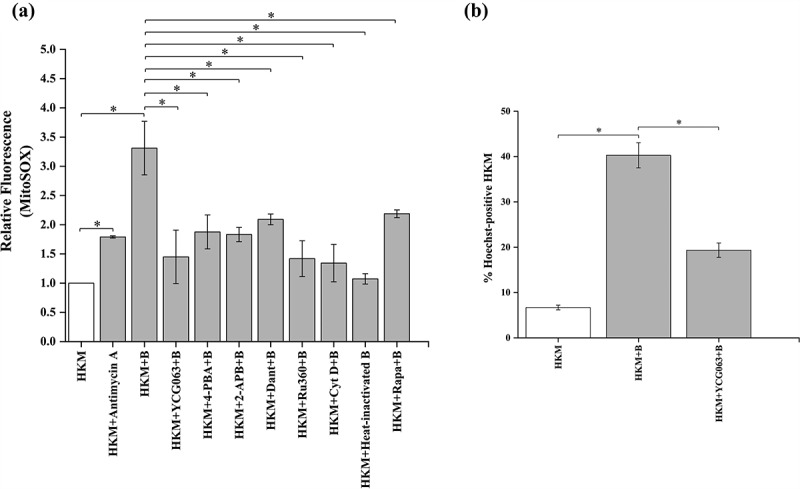
***A. hydrophila* induces pro-apoptotic mtROS production**. HKM pre-incubated with Antimycin A, YCG063, 4-PBA, 2-APB, Dant, Ru360, Cyt D, heat-killed bacteria and Rapa were infected with or without *A. hydrophila* and (a) changes in mtROS levels were studied at 4 h p.i. and (b) HKM apoptosis was studied at 24 h p.i. Vertical bars represent mean ± SE (n = 3). Asterisk indicates significant difference between indicated groups (*p < 0.05). HKM, uninfected HKM; HKM+B, HKM infected with *A. hydrophila*; HKM+YCG063 + B, HKM pre-incubated with YCG063 infected with *A. hydrophila*; HKM+4-PBA+B, HKM pre-incubated with 4-PBA infected with *A. hydrophila*; HKM+2-APB +B, HKM pre-incubated with 2-APB infected with *A. hydrophila*; HKM+Dant+B, HKM pre-incubated with dantrolene infected with *A. hydrophila*; HKM+Ru360 + B, HKM pre-incubated with Ru360 infected with *A. hydrophila*; HKM+Cyt D + B, HKM pre-incubated with Cyt D infected with *A. hydrophila*; HKM+heat-killed B, HKM infected with heat-killed *A. hydrophila*; HKM+Rapa+B, HKM pre-incubated with rapamycin infected with *A. hydrophila*, HKM+Antimycin A, HKM pre-incubated with Antimycin A.

Mitochondrial ROS (mtROS) plays an important role in microbial pathogenesis and host defense [47,48]. However, the role of mtROS has not been studied in *A. hydrophila*-pathogenesis. To look into this, HKMs were infected with *A. hydrophila* and mtROS production monitored at indicated time intervals p.i. We observed maximum mtROS production at 4 h p.i. (Fig S2) and choose this time interval for further studies. Dead (Heat-killed) *A. hydrophila* failed to induce mtROS production (Figure 3a). Electron transport chain (ETC) inhibitor antimycin A induces mtROS [49] and was used as positive control in the study (Figure 3a). Parallelly, HKM pre-incubated with mtROS inhibitor YCG063 were infected with *A. hydrophila* and apoptotic cell death studied at 24 h p.i. We observed pre-treatment with YCG063 alleviated HKM apoptosis (Figure 3b) suggesting mtROS production is critical in *A. hydrophila* pathogenesis.

(Ca^2+^)_ER_ is released through membrane resident IP3R and RyR channels, which gets mobilized into the mitochondria influencing mtROS generation [15,22]. To study this, HKMs pre-incubated with IP3R and RyR antagonist 2-APB and dantrolene (Dant), respectively, were infected with *A. hydrophila* and the changes in mtROS levels monitored at 4 h p.i. We observed significant reduction in mtROS levels in the presence of both 2-APB and Dant in *A. hydrophila-*infected HKM (Figure 3a). In parallel study, HKM was pre-incubated with 4-PBA, and the changes in *A. hydrophila*-induced mtROS production measured at 4 h p.i. We observed that alleviating ER-stress attenuated mtROS production in *A. hydrophila*-infected HKM (Figure 3a). These results established the importance of ER-stress and ensuing (Ca^2+^)_ER_ dynamics on mtROS generation in *A. hydrophila-*infected HKM.

Our previous studies suggested the proximity between mitochondria and ER facilitate the uptake of (Ca^2+^)_ER_ by mitochondria [3]. We reasoned that if the proximity between the two organelles is prevented, (Ca^2+^)_ER_ mobilization would be interrupted with a concomitant decline in mtROS levels. To study this, HKMs were pre-incubated with Cyt D which inhibits mitochondrial motility [3,50], then infected with *A. hydrophila* and the changes in mtROS production monitored at 4 h p.i. We observed significant reduction in mtROS production in the presence of Cyt D (Figure 3a), that clearly established co-localization of ER and mitochondria is important for the uptake of (Ca^2+^)_ER_ and mtROS generation in *A. hydrophila*-infected HKM.

We followed this by identifying the molecular mechanism of (Ca^2+^)_ER_ dynamics. Mitochondrial calcium uniporters (MCU) present on the mitochondrial membrane facilitates the uptake of (Ca^2+^)_ER_ into the mitochondria [51]. Additionally, HKMs were pre-incubated with Ru360, a selective inhibitor of MCU [17], and measured mtROS levels at 4 h p.i. Pre-treatment with Ru360 led to significant decline in mtROS production in the infected HKM (Figure 3a), suggesting MCU to be responsible for regulating (Ca^2+^)_ER_ influx in mitochondria.

We were interested to know whether mtROS *per se* has any role in the induction of ER-stress in *A. hydrophila*-infected HKM. Toward that end, HKMs were pre-incubated with mtROS inhibitor YCG063 [52], and then infected with *A. hydrophila* and *chop* expression studied by RT-qPCR at 2 h p.i. We noted that pre-treatment with YCG063 significantly repressed *chop* expression in the infected HKM (Figure 1). Additionally, antimycin A treatment also upregulated *chop*-mRNA expression in HKM (Figure 1). These results collectively implicated that the interplay between (Ca^2+^)_ER_ and mtROS signaling is critical for *A. hydrophila* pathogenesis.

### *A*. *hydrophila* inhibits mitophagy triggering mtDNA release into cytosol

**Figure 4.**
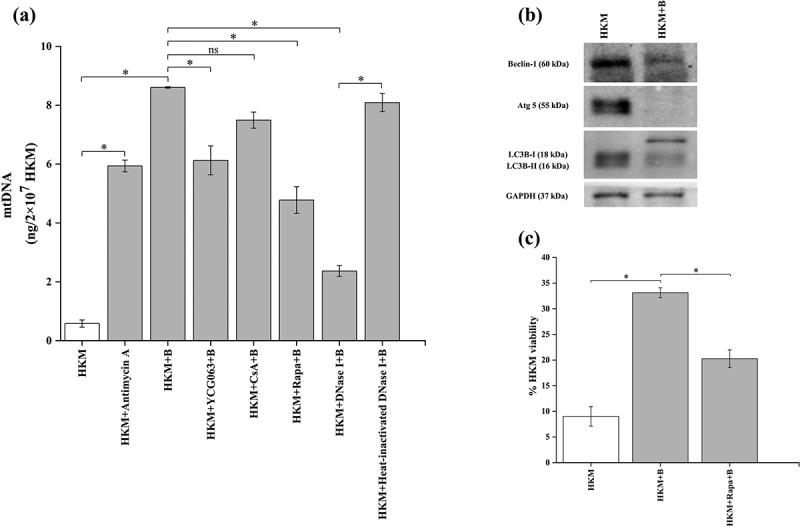
***A. hydrophila* triggers translocation of mtDNA *via* suppression of autophagy**. (a) HKM were pre-incubated with Antimycin A, YCG063, CsA, Rapa, or transfected with DNase I and heat-inactivated DNase I were infected with or without *A. hydrophila* and presence of mtDNA in cytosol was studied at 1 h p.i. (b) HKMs were infected with *A. hydrophila* and expression of autophagic proteins (beclin-1, atg 5, and LC3B) were studied at by immunoblotting. (c) HKM incubated with rapamycin infected with *A. hydrophila* and percentage HKM viability was studied at 24 h p.i. using trypan blue dye exclusion method. Vertical bars represent mean ± SE (n = 3). Asterisk indicates significant difference between indicated groups (*p < 0.05). ns indicates not significant between indicated groups. HKM, uninfected HKM; HKM+B, HKM infected with *A. hydrophila*; HKM+YCG063 + B, HKM pre-incubated with YCG063 infected with *A. hydrophila*; HKM+CsA+B, HKM pre-incubated with Cyclosporin A infected with *A. hydrophila*; HKM+Rapa+B, HKM pre-incubated with rapamycin infected with *A. hydrophila*; HKM+DNase I + B, HKM transfected with DNase I infected with *A. hydrophila*; HKM+heat-inactivated DNase I + B, HKM transfected with heat-inactivated DNase I infected with *A. hydrophila*; HKM+Antimycin A, HKM pre-incubated with Antimycin A.

It has been observed that mtROS causes irreversible damage to mitochondria triggering the release of mitochondrial DNA (mtDNA) [27]. First, we investigated the cytosolic translocation of mtDNA in *A. hydrophila* pathogenesis. HKMs were infected with *A. hydrophila* and the cytosolic migration of mtDNA studied. The mito-genome of *C. gariepinus* has been sequenced [53] and using that information specific primers for the mitochondrial gene, *cox2* of *C. gariepinus* was designed (Table 2). HKMs were lysed at indicated time intervals, mtDNA was isolated from the cytosol, PCR amplified using *cox2* primers and the presence of cytosolic mtDNA studied. Our results suggested the presence of mtDNA in cytosol of infected HKM at 1 h p.i., (Fig S3); therefore, this time point was selected for further experiments. We hypothesized that *A. hydrophila*-induced mtROS triggers the release of mtDNA into cytosol, thereby impacting host immunity. For this, HKMs were pre-incubated with YCG063 following *A. hydrophila* infection. At 1 h p.i., mtDNA was quantitated using RT-PCR and the amount of cytosolic mtDNA (absolute quantification) was interpolated from the standard curve (see M&M section). Our results suggested inhibition of mtROS attenuated mtDNA release into cytosol (Figure 4a). Furthermore, treatment with antimycin A increased the amount of cytosolic mtDNA in *A. hydrophila*-infected HKM (Figure 4a). These results confirm previous findings suggesting the role of mtROS on releasing mtDNA in cytosol [27].

**Table 2.** List of RT-qPCR primer sequences

Gene	Primer sequences
*il1b*	FP: 5ʹ- CCACAGAGTTTAGTGACCAGGAG −3ʹ RP: 5ʹ-ACCTTGTCTTGCAGGCTGTAG −3’
*inos*	FP: 5ʹ- GACCATCACAGACCACCACA −3ʹ RP: 5ʹ- GACATAGGAGGTACCAGCCAA −3’
*cox2*	FP: 5ʹ-TAATCCCAACACAAGACCTTGCACC-3ʹ RP: 5ʹ-GAAGGATGTTTGGTTTAATCGTCCTGG-3’
*chop*	FP: 5ʹ- GTTGGAGGCGTGGTATGAAG −3ʹ RP: 5ʹ – GAAACTCCGGCTCTTTCTCG – 3’
*β–act*	FP: 5ʹ-CGAGCAGGAGATGGGAACC-3ʹ RP: 5ʹ-CAACGGAAACGCTCATTGC-3’

The next step was exploring the mechanisms underlying release of mtDNA into cytosol and studying its impact on *A. hydrophila*-infected HKM. It has been suggested that mitochondrial pore transition (MPT) facilitates the release of mtDNA into the cytosol [27]. We observed that pre-incubation with MPT inhibitor CsA failed to inhibit the cytosolic migration of mtDNA in *A. hydrophila*-infected HKM (Figure 4a). Autophagy proteins have also been implicated in regulating the cytosolic migration of mtDNA [27]. To investigate this, HKMs were infected with *A. hydrophila* and the expression of autophagy proteins Beclin-1, LC3B and Atg 5 studied by immunoblot. We observed down-regulation of Beclin-1, LC3B and Atg 5 expression in *A. hydrophila*-infected HKM (Figure 4b). Additionally, HKMs were pre-incubated with autophagy inducer, rapamycin and then infected with *A. hydrophila* and cytosolic translocation of mtDNA studied at 1 h p.i. and HKM viability monitored at 24 h p.i. respectively. We observed rapamycin inhibited the cytosolic translocation of mtDNA (Figure 4a) with concomitant reduction in HKM death (Figure 4c). These findings clearly suggested that *A. hydrophila* inhibits mitophagy facilitating the release of mtDNA in cytosol, thereby triggering HKM death.

### mtDNA triggers pro-inflammatory caspase-1/IL-1β cascade in *A.* *hydrophila*-infected HKM

**Figure 5.**
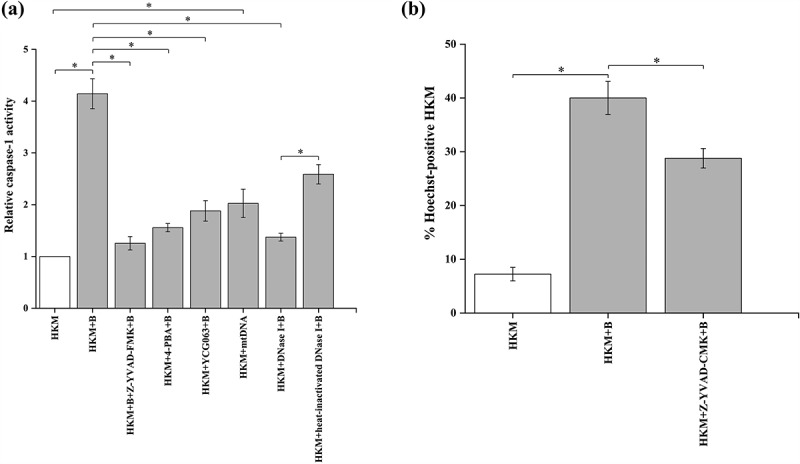
**Cytosolic mtDNA triggers activation of pro-apoptotic caspase-1 in *A. hydrophila*-infected HKM**. (a) HKMs pre-incubated with Z-YVAD-FMK, 4-PBA, YCG063 or transfected with mtDNA, DNase I, heat-inactivated DNase I were infected with or without *A. hydrophila* and caspase-1 activity studied at 12 h p.i. (b) HKMs incubated with Z-YVAD-FMK were infected with *A. hydrophila* and HKM apoptosis studied at 24 h p.i. Vertical bars represent mean ± SE (n = 3). Asterisk indicates significant difference between indicated groups (*p < 0.05). HKM, uninfected HKM; HKM+B, HKM infected with *A. hydrophila*; HKM+Z-YVAD-FMK+B, HKM pre-incubated with Z-YVAD-FMK infected with *A. hydrophila*; HKM+4-PBA+B, HKM pre-incubated with 4-PBA infected with *A. hydrophila*; HKM+YCG063 + B, HKM pre-incubated with YCG063 infected with *A. hydrophila*; HKM+mtDNA, HKM transfected with mtDNA; HKM+DNase I + B, HKM transfected with DNase I infected with *A. hydrophila*; HKM+heat-inactivated DNase I + B, HKM transfected with heat-inactivated DNase I infected with *A. hydrophila.*

**Figure 6.**
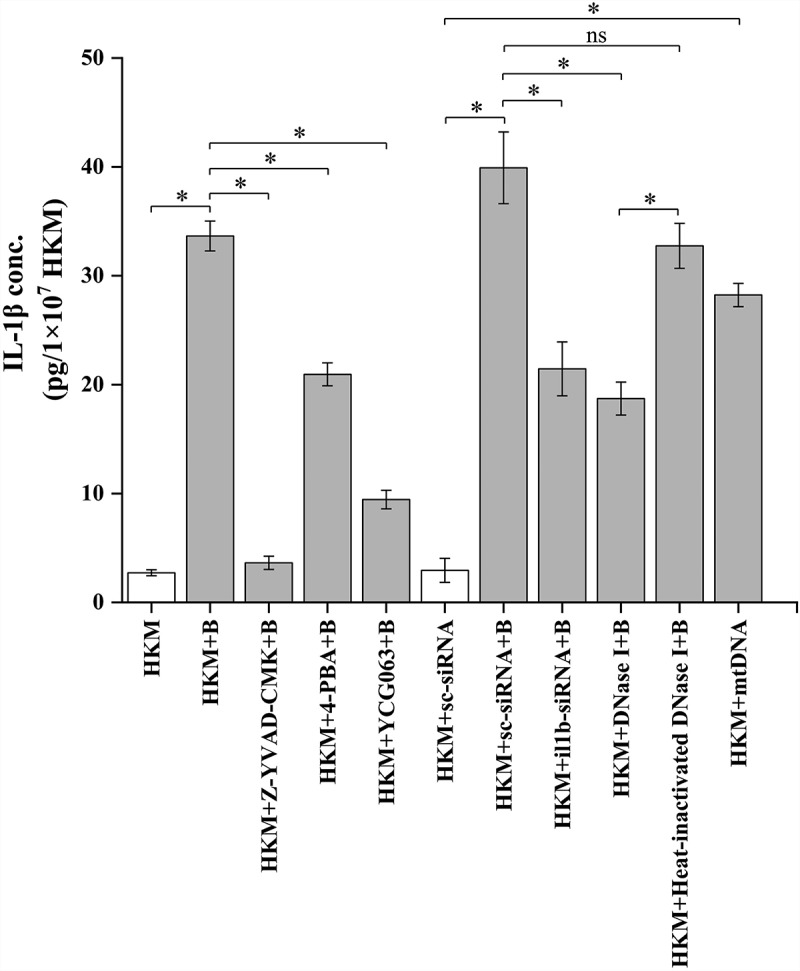
***A. hydrophila*-induced mtDNA/caspase-1 axis triggers IL-1β production**. HKM pre-incubated with Z-YVAD-FMK, 4-PBA, YCG063 or transfected with sc-siRNA, *il1b*-siRNA, DNase I, heat-inactivated DNase I, mtDNA were infected with or without *A. hydrophila* and production of IL-1β protein was measured at 24 h p.i. Vertical bars represent mean ± SE (n = 3). Asterisk indicates significant difference between indicated groups (*p < 0.05). ns indicates not significant between indicated groups. HKM, uninfected HKM; HKM+B, HKM infected with *A. hydrophila*; HKM+Z-YVAD-FMK+B, HKM pre-incubated with Z-YVAD-FMK infected with *A. hydrophila*; HKM+4-PBA+B, HKM pre-incubated with 4-PBA infected with *A. hydrophila*; HKM+YCG063 + B, HKM pre-incubated with YCG063 infected with *A. hydrophila*; HKM+sc-siRNA, HKM transfected with sc-siRNA; HKM+sc-siRNA+B, HKM transfected with sc-siRNA infected with *A. hydrophila*, HKM+*il1b*-siRNA+B, HKM transfected with *il1b*-siRNA infected with *A. hydrophila*; HKM+DNase I + B, HKM transfected with DNase I infected with *A. hydrophila*; HKM+heat-inactivated DNase I + B, HKM transfected with heat-inactivated DNase I infected with *A. hydrophila*; HKM+mtDNA, HKM transfected with mtDNA.

We next explored how mtDNA impacts *A. hydrophila* pathogenesis. The role of mtDNA in activating pro-inflammatory caspase-1/IL-1β cascade is well established [27]. This encouraged us to evaluate the role of mtDNA in activating the caspase-1/IL-1β axis in *A. hydrophila*-infected HKM. At the outset, HKMs were infected with *A. hydrophila* and the changes in caspase-1 activity and IL-1β levels measured. We observed maximum caspase-1 activity at 12 h p.i. and peak IL-1β levels at 24 h p.i. respectively and choose these time points for further studies [54]. To correlate caspase-1 activation with *A. hydrophila*-pathogenesis, HKMs were pre-incubated with caspase-1 inhibitor, Z-YVAD-FMK and then infected with *A. hydrophila* and caspase-1 activity and HKM apoptosis studied. Pre-treatment with Z-YVAD-FMK inhibited caspase-1 activity (Figure 5a) and HKM apoptosis (Figure 5b) implicating the role of caspase-1 in *A. hydrophila*-pathogenesis. Subsequently, HKM pre-incubated with Z-YVAD-FMK were infected with *A. hydrophila* and IL-1β levels measured. We observed that inhibiting caspase-1 activity interfered with IL-1β production in the infected HKM (Figure 6).

Our next step was elucidating the connection between ER-stress/mtROS axis and caspase-1/IL-1β cascade. HKMs were pre-incubated separately with 4-PBA and YCG063, respectively, then infected with *A. hydrophila* and caspase-1 activity and IL-1β levels studied. We observed that pre-incubation with 4-PBA and YCG063 inhibited caspase-1 (Figure 5a) and suppressed IL-1β levels (Figure 6) in *A. hydrophila*-infected HKM. Collectively, these findings signify the role of ER-stress-induced mtROS in triggering caspase-1/IL-1β cascade activation in *A. hydrophila*-infected HKM.

We followed this by transfecting mtDNA and studied the effect on caspase-1 and IL-1β. It was observed that transfection of mtDNA augmented caspase-1 activation (Figure 5a) and IL-1β production (Figure 6) in *A. hydrophila* infected HKM. To confirm the role of mtDNA in activating the caspase-1/IL-1β axis, HKMs were transfected with DNase I and then infected with *A. hydrophila*. We observed that DNase I transfection interfered with the cytosolic accumulation of mtDNA and inhibited caspase-1 activity and IL-1β production in the infected HKM. The transfection of heat-inactivated DNase I failed to reverse the translocation of mtDNA and inhibit caspase-1 activity in *A. hydrophila-*infected HKM (Figure 5a). To this, we concluded that cytosolic mtDNA triggers the caspase-1/IL-1β cascade in *A. hydrophila-*infected HKM, thereby influencing the pathogenesis induced by the bacterium in fish.

### IL-1β triggers pro-apoptotic NO in *A.* *hydrophila*-infected HKM

**Figure 7.**
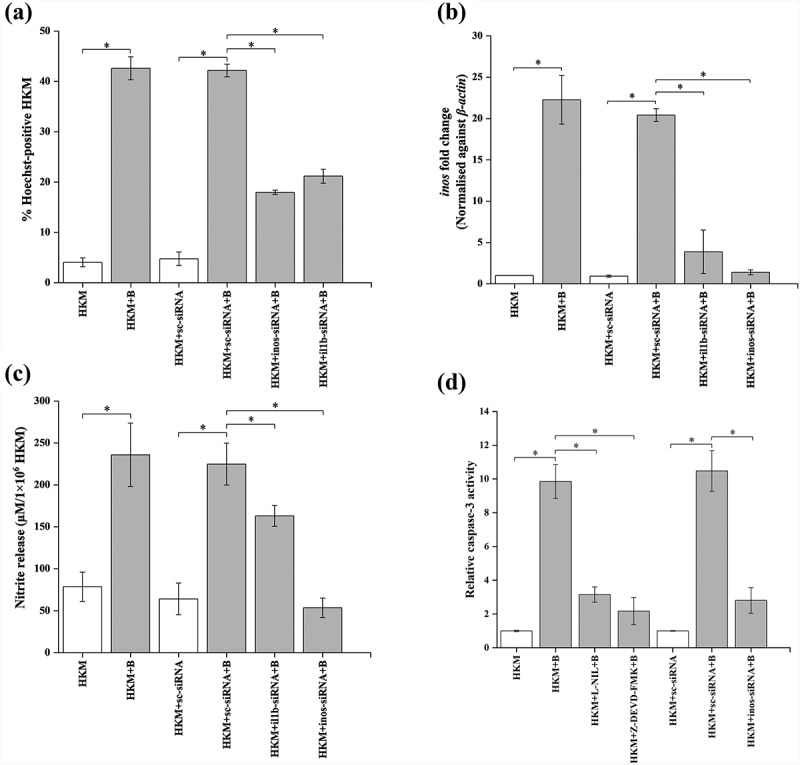
**IL-1β induces activation of pro-apoptotic *inos*/NO axis in *A. hydrophila*-infected HKM**. HKM transfected with sc-siRNA, *il1b*-siRNA, *inos*-siRNA or pre-incubated with L-NIL, Z-DEVD-FMK and infected with *A. hydrophila* and at 24 h p.i., (a) HKM apoptosis, (b) the expression of *inos* mRNA was studied, (c) nitrite levels were measured, and (d) caspase-3 activity was studied at 24 h p.i. Vertical bars represent mean ± SE (n = 3). Asterisk indicates significant difference between indicated groups (*p < 0.05). HKM, uninfected HKM; HKM+B, HKM infected with *A. hydrophila*; HKM+L-NIL+B; HKM pre-incubated with L-NIL and infected with *A. hydrophila*; HKM+Z-DEVD-FMK+B, HKM pre-incubated with Z-DEVD-FMK and infected with *A. hydrophila*; HKM+sc-siRNA, HKM transfected with sc-siRNA; HKM+sc-siRNA+B, HKM transfected with sc-siRNA infected with *A. hydrophila*; HKM+*il1b*-siRNA+B, HKM transfected with *il1b*-siRNA infected with *A. hydrophila*; HKM+*inos*-siRNA+B, HKM transfected with *inos*-siRNA infected with *A. hydrophila.*

Previous studies suggested that IL-1β triggers necrosis [55] and apoptosis [56] under various conditions of stress. To investigate this, HKMs were transfected with *il1b*-siRNA and apoptosis studied at 24 h p.i. We noted significant decline in the number of Hoechst positive HKM (Figure 7a) suggesting pro-apoptotic role of IL-1β in *A. hydrophila* pathogenesis. We wondered how IL-1β contributed toward *A. hydrophila*-induced HKM apoptosis. Several reports have implicated the contribution of NO in IL-1β mediated apoptosis [57,58]. We observed time dependent increase in *inos*-mRNA expression (Fig S4) and NO levels recorded at 24 h p.i [3]. In view of this, HKM transfected with *il1b*-siRNA were infected with *A. hydrophila* and the changes in *inos* expression and NO levels monitored at 24 h p.i. It is clear from RNAi studies that there was marked reduction in *inos* expression (Figure 7b) and NO production (Figure 7c) with parallel decrease in HKM apoptosis (Figure 7a) and caspase-3 activity (Figure 7d) which suggested positive correlation between IL-1β production and NO-mediated apoptosis of *A. hydrophila*-infected HKM.

## Discussion

*A. hydrophila* displays wide host tropism that includes both poikilothermic and homoeothermic organisms, including mammals. How the bacterium infects such a wide range of hosts is still not known. Therefore, to have an insight of the molecular underpinning of *A. hydrophila*-pathogenesis we selected HKM as a model. Fish can be considered a good model for the present study because *A. hydrophila* is a natural fish pathogen [19] and significant similarity between fish and mammalian immune systems [59]. Studying *A. hydrophila*-HKM interactive pathways in fish will shed important light on the universal aspects of these regulations, beyond the fish syndrome which otherwise may not possible in other systems such as mammals.

Our previous studies suggested that *A. hydrophila*-induced alterations in (Ca^2+^)c levels is critical toward inducing ER-stress [3]. We also observed that the PI3K/PLC axis plays an important role in inducing (Ca^2+^)c alterations in *A. hydrophila*-infected HKM [14]. The two independent findings encouraged us to hypothesize a positive correlation between PI3K/PLC axis and ER-stress in *A. hydrophila*-pathogenesis. Toward that end, we studied the expression of well-known apoptotic ER-stress marker, CHOP in the absence of PI3K/PLC signaling. The marked decline in CHOP expression implicates PI3K/PLC mediated release of (Ca^2+^)c is indispensable for ER-stress in *A. hydrophila*-infected HKM. PI3K is well known to favor cell survival *via* activation of downstream kinase, Akt/protein kinase B. The involvement of PI3K in ER-stress induced apoptotic cascade is interesting and prompted us to study the underlying molecular mechanisms. Previous studies suggested that *A. hydrophila* suppresses Akt activation [60], and Akt inhibition triggers apoptosis [61]. The role of Akt in ER-stress is not well understood and we reasoned that prolonged ER-stress suppresses the activation of Akt, in *A. hydrophila*-infected HKM. We observed suppressed Akt activity in the infected HKM, which got reversed on ameliorating ER-stress leading to increased HKM survival. Our findings are in concordance with previous findings [62] and suggest the central role of CHOP-Akt crosstalk in *A. hydrophila* pathogenesis.

We wondered how ER-stress inhibits Akt phosphorylation to promote HKM apoptosis. MAPK family members have been implicated in apoptosis and it has been suggested that CHOP inactivates Akt phosphorylation by activating MAPK [63]. We observed a proportionate increase in phospho-JNK expression with apoptotic implication in *A. hydrophila*-infected HKM which was reduced in *chop*-knockdown HKM suggesting a positive relation between ER-stress and JNK activation. Concordantly, repressing JNK activation also resulted in increased Akt activity and HKM viability. Our results for the first time suggested that CHOP promotes apoptosis of *A. hydrophila*-infected HKM by inhibiting AKT activation and enhancing MAPK signaling.

ER-stress-induced mitochondrial dysfunction has a prominent role in triggering apoptosis of *A. hydrophila*-infected HKM [3], but the molecular intermediates responsible for the process has not been addressed. Under stress, ER releases (Ca^2+^)_ER_, which is taken up by the mitochondria triggering the production of mtROS [22,64]. mtROS has not been implicated in *A. hydrophila* pathogenesis. Therefore, we evaluated mtROS production in *A. hydrophila*-infected HKM and observed significant mtROS generation which was inhibited on ameliorating ER-stress. Additionally, pre-treatment with (Ca^2+^)_ER_ release modulators and MCU inhibitors also repressed mtROS levels altogether confirming the positive correlation between (Ca^2+^)_ER_ and mtROS production in *A. hydrophila*-infected cells. Mitochondrial-Ca^2+^ uptake is expedited by the proximity between the mitochondria and ER [3,65]. The reduction in mtROS levels on pre-treatment with cyt D, which inhibits mitochondrial movement toward ER lends support to the hypothesis. We are presently trying to ascertain the molecular intermediates involved in ER-mitochondria cross-talk.

mtROS generated under varied conditions of stress is known for its ability to induce apoptosis [66,67], but the same has not been reported in fish. Our next step was correlating mtROS with *A. hydrophila*-induced HKM apoptosis and for that, we used mtROS augmenter (antimycin A) and inhibitor (YCG063). Interestingly, our results revealed pro-apoptotic implication of mtROS in *A. hydrophila*-infected HKM. This is the first report on the role of mtROS impacting microbial-pathogenesis in fish to the best of our knowledge. With the consensus of previous reports [66,67] and our findings, we posit that the potential of mtROS to induce apoptosis is conserved across species. Collectively, our findings lend support to earlier studies suggesting that the close connection between ER and mitochondria, and ensuing Ca^2+^ dynamics between them determine cell-fate, leading to different pathological conditions [3,68,69].

We further aimed to study how mtROS influence HKM apoptosis. Increased mtROS levels affect several cellular targets including the organelle itself with pathological consequences [70,71]. mtROS induced structural-functional alterations in mitochondria release mtDNA into the cytosol [27]. mtDNA is a DAMP which stimulates innate immune responses that influence antimicrobial responses, inflammatory pathology, and apoptosis [25,26]. We had previously observed *A. hydrophila-*induced structural-functional alterations in mitochondria of HKM [3]. We hypothesized that elevated mtROS triggers the cytoplasmic migration of mtDNA thereby contributing in *A. hydrophila* pathogenesis. Indeed, our results confirmed that mtROS facilitates the cytosolic accumulation of mtDNA in infected HKM. Additionally, inhibiting the cytosolic accumulation of mtDNA attenuated HKM death which clearly established the pro-apoptotic implication of cytosolic mtDNA in *A. hydrophila* pathogenesis.

The mechanism underlying mtDNA escape to the cytosol is still not well understood [24,72]. MPT is observed to play a vital role in mitochondrial-pathology and previous studies have implicated its role in release of mtDNA in the cytosol [27]. We could not observe significant role of MPT on the translocation of mtDNA in infected HKM. Several mechanisms have been suggested to be involved in the release of mtDNA [71–73] and we are trying to ascertain the role of other factors in triggering mtDNA release in *A. hydrophila*-infected cells. Nonetheless, this is the first study linking mtDNA in *A. hydrophila* pathogenesis. Our results are of great significance as mtDNA is a critical innate immune agonist that impacts host responses.

Autophagy helps in the removal of damaged and malfunctioned cellular components [74,75]. It is also increasingly evident that autophagy is intimately associated with conferring innate protection against microbial pathogens [76]. Earlier studies suggest, suppressed autophagy promotes mtROS generation and the cytosolic translocation of mtDNA [27,77,78]. We observed suppressed expression of autophagic proteins in *A. hydrophila*-infected HKM. Furthermore, treatment of HKM with autophagy inducer, rapamycin resulted in the decreased accumulation of cytosolic mtDNA and inhibited HKM death. To this, we concluded that impaired mitophagy has a major role in *A. hydrophila* pathogenesis. The mechanism by which autophagy is suppressed in *A. hydrophila*-infected HKM is important to understand the pathogenesis of UDS.

The involvement of mtROS/mtDNA axis has been well elucidated as a platform for inflammasome activation in mammals [76,77]; however, the same has not been explained in fishes. The structure and function of Nod-like receptors (NLRs), the main component of inflammasome formation, are also poorly understood in fish. One important outcome of inflammasome is caspase-1/IL-1β activation [77,79]. The involvement of caspase-1/ IL-1β axis has been described earlier in several fish species [32,34]. Moreover, *A. hydrophila* related toxins induce caspase-1/IL-1β activation in bone marrow-derived macrophages of mice [80], but their functional consequences are not completely known in fish. We noted enhanced caspase-1 activity in *A. hydrophila*-infected HKM was repressed on ameliorating ER-stress and mtROS production. This evidently indicated the ER-stress induced mtROS production is inevitable for caspase-1 activation in *A. hydrophila-*infected HKM.

An interesting outcome of this study was the pro-active role of cytosolic mtDNA in triggering caspase-1/IL-1β activity. Transfection of DNase I prevented the cytosolic aggregation of mtDNA and inhibited the activation of caspase-1/IL-1β axis in *A. hydrophila*-infected HKM. The role of cytosolic DNA (exogenous) has been reported in caspase-1 activation in several model systems [81,82]. Additionally, the role of mtDNA in caspase-1/IL-1β cascade has also been observed in mammalian cells under different conditions of stress [27,83]. This is the first study implicating mtDNA in the activation of caspase-1/IL-1β cascade and regulating microbial pathogenesis in fish. Based on these findings and earlier reports, we speculate this to be an evolutionary conserved host response against microbial pathogens.

We further monitored how IL-1β induce apoptosis of the infected HKM? IL-1β is mainly responsible for generating a pro-inflammatory response during host-pathogen interaction [84], and there are also reports suggesting its involvement in apoptosis [56]. However, there are reports documenting, IL-1β induces pro-apoptotic effects by affecting NO production [57]. We had previously reported that NO primarily contributed in the activation of extrinsic pathway of apoptosis in *A. hydrophila*-infected HKM [3]. Our RNAi studies indeed demonstrated marked inhibition in *inos*-mRNA and NO levels with concomitant decline in apoptosis of *A. hydrophila*-infected HKM, in absence of IL-1β. To this, we concluded that the caspase-1/IL-1β cascade culminates in NO-mediated activation of extrinsic caspases, triggering the apoptosis of *A. hydrophila*-infected HKM. Together, our results reveal an alternate pathway of caspase activation in *A. hydrophila*-pathogenesis.

**Figure 8.**
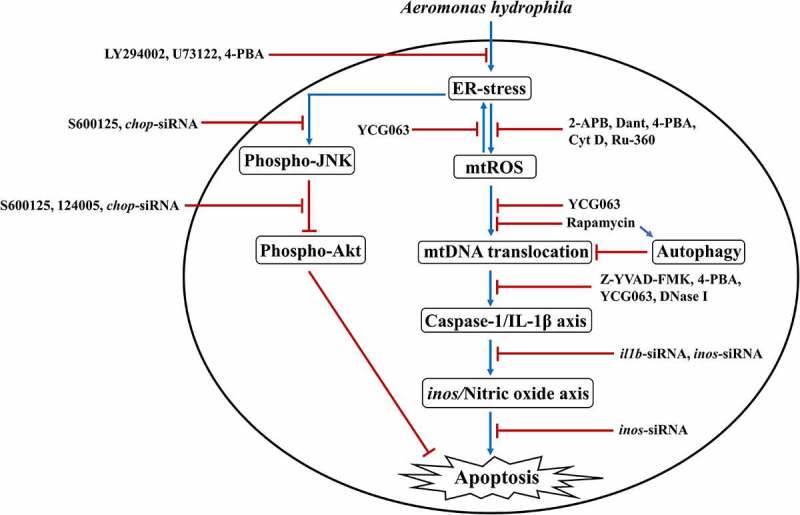
**Overview of the study**. *A. hydrophila*-induced activation of PI3K/PLC axis triggers ER-stress leading to downstream phosphorylation of JNK and consequent inhibition of Akt phosphorylation. ER-stress instigates mtROS production leading to cytosolic translocation of mtDNA *via* suppression of autophagy. The cytosolic mtDNA activates inflammatory caspase-1/IL-1β axis leading to NO-mediated apoptosis of *A. hydrophila*-infected HKM.

In this study, we have shown that PI3K-PLC signaling induced (Ca^2+^)_C_ efflux results in the activation of pro-apoptotic ER-stress *via* modulation of Akt-JNK signaling pathway in *A. hydrophila*-infected HKM. Additionally, our observations demonstrated that interplay between (Ca^2+^)_ER_ dynamics and mtROS signaling contributes to the cytosolic translocation of mtDNA. Based on our findings, we propose that impairment in autophagic machinery favors accretion of impaired mitochondria which release mtDNA in cytosol, amplifying the activation of inflammatory caspase-1/IL-1β signalosome and consequently NO-mediated HKM apoptosis (Figure 8). Our findings would be helpful toward understanding *A. hydrophila* pathogenesis and controlling UDS.

## Materials and methods

### Animal maintenance

All the catfish (*Clarias gariepinus*) studies were carried out listed in the procedures issued by Committee for the purpose of Control and Supervision of Experiments on Animals (CPCSEA), Govt. of India and permitted by Animal Ethics Committee (DU/ZOOL/IAEC-R/2013/33), University of Delhi. Prior to experiments, *C. gariepinus* were acclimatized for 15 days and fed with chicken liver *ad libitum* [19].

### HKM isolation, infection with *A.* *hydrophila* and inhibitor studies

*C. gariepinus* and *A. hydrophila* (Strain 500,297) were used in the study. The protocols for HKM isolation, and infection of HKM with *A. hydrophila* (MOI 1:50) have been described earlier [14].

zHKMs were incubated separately with ER stress inhibitor (4-PBA, 10 µM, Sigma), mtROS inhibitor (YCG063, 10 µM, Calbiochem), ETC inhibitor (antimycin A, 50 µM, Sigma), MPTP inhibitor (Cyclosporin A, 5 µM, Sigma), RyR inhibitor (Dantrolene, 10 µM, Sigma), Cytochalasin D (Cyt D, 5 µg/mL, Sigma), Autophagy inducer (Rapamycin, 20 µM, Sigma), Thapsigargin (1 µM, Sigma), Akt inhibitor (124,005, 10 µM, Calbiochem) for 1 h and mitochondrial Ca^2+^ uptake inhibitor (Ru360, 10 µM, Calbiochem), Caspase-1 inhibitor (Z-YVAD-FMK, 7.5 µM, Biovision), Caspase-3 inhibitor (Z-DEVD-FMK, 10 µM, Biovision), IP3R inhibitor (2-APB, 100 μM, Sigma), PLC inhibitor (U73122, 2 μM, Enzo Life Science), PI3-Kinase inhibitor (LY-294002 hydrochloride, 14.5 μM, Sigma), intracellular Ca^2+^chelator, (BAPTA-AM, 5 mM, Sigma), and JNK inhibitor (SP600125, 10 µM, Calbiochem) for 2 h followed by *A. hydrophila* infection as mentioned earlier [3]. The inhibitor concentrations had no adverse effects on HKM viability and bacterial growth (data not shown).

### siRNA transfection

The protocol for transfecting HKM with specific or scrambled siRNA (sc-siRNA) (Table 1) has been described earlier [14]. Silencing of genes was checked by RT-qPCR and HKM viability monitored throughout the experiment.

**Table 1.** List of siRNA sequences

Gene	siRNA sequences
*il1b*	Sense: 5ʹ-GAAACUCACUGAAAGAGUU-3ʹ Antisense: 5ʹ-AACUCUUUCAGUGAGUUUC-3’
*inos*	Sense: 5ʹ-CGCUACAACAUUCUUGAGA-3ʹ Antisense: 5ʹ-UCUCAAGAAUGUUGUAGCG-3’

### mtROS production

mtROS was monitored using the MitoSOX™ Red mitochondrial superoxide indicator (Molecular Probes) [85]. HKMs (1 × 10^6^/mL) were incubated with inhibitors and infected with *A. hydrophila*. HKMs were washed, incubated with MitoSOX (5 μM) for 20 min and the fluorescence levels measured at A_510nm_ excitation and A_580nm_ emission (Molecular Devices, Spectramax).

### Akt assay

HKMs (1 × 10^7^ /mL) were incubated with inhibitors or transfected with *chop*-siRNA and infected with *A. hydrophila*. The total Akt and phospho-Akt levels in the cell lysates were measured with Duoset IC ELISA kit (R&D Systems) following the instructions specified by the manufacturer.

### mtDNA isolation, detection, and transfection

mtDNA from cytosol was isolated as described [27]. HKMs (1 × 10^7/^mL) incubated with inhibitors were infected with *A. hydrophila*. HKMs were harvested at different time points, lysed, and homogenized in 100 mM Tricine-NaOH (pH 7.4) supplemented with sucrose (0.25 M), EDTA (1 mM), proteinase K (5 mg/mL) and centrifuged at 700 × g. Next, the supernatant was centrifuged at 10,000 × g at 4°C for 30 min. mtDNA was obtained from the supernatant (cytosolic fraction) using DNeasy Blood & Tissue Kit (Qiagen) according to the manufacturer’s instructions. The supernatant was mixed and incubated with Buffer AL (200 µL) at 56°C for 10 min. Ethanol (200 µL) was added and centrifuged at 6000 × g for 1 min. Then, Buffer AW1 (500 µL) was added and centrifuged at 6000 × g for 1 min. Flow-through was discarded and Buffer AW2 (500 µL) was added and centrifuged at 20,000 × g for 3 min. mtDNA was eluted using Buffer AE (200 µL), incubated at RT for 1 min, and then centrifuge for 1 min at 6000 × g. The presence of mtDNA in cytosolic fraction was confirmed by PCR using mitochondrial DNA encoded gene *cox2* specific primers.

mtDNA (50 ng) was transfected using HiPerFect Transfection reagent (Qiagen). The HiPerFect-mtDNA complex was incubated and the complex gently introduced to the HKM. The transfected HKMs were incubated for 6 h at 30°C, washed and proceeded for subsequent studies. Transfection of mtDNA was confirmed as described above.

### Immunoblot

HKMs (1 × 10^7/^mL) incubated with inhibitors or transfected with sc-siRNA or *chop*-siRNA were infected with *A. hydrophila*. The HKMs were lysed for CHOP at 2 h p.i., LC3B, Beclin-1, Atg 5 at 4 h p.i. and for total and phospho-JNK at 24 h p.i., resolved on SDS-PAGE and transferred to PVDF membrane. Membrane was blocked with 1% BSA in TBST for 1 h and subsequently incubated with anti-CHOP (1: 100, Cell Signaling Technology), anti-LC3B (1: 1000, Abcam), anti-Beclin-1 (1: 1000, Abcam), anti-Atg 5 (1: 1000, Abcam) and anti-total JNK and anti-phosphorylated JNK (Cell Signaling Technology, 1: 500 dilution), respectively, overnight at 4°C. Anti-GAPDH antibody (Santacruz, 1: 5000) was used for the normalization of Beclin-1, Atg 5, LC3B, and anti-β-actin antibody (Cell Signaling Technology, 1:10,000 dilution) was used for CHOP and JNK.

### Quantitative real time PCR (RT-qPCR)

HKMs (1 × 10^7/^mL) incubated with inhibitors or transfected with sc-siRNA or *chop*-siRNA, *il1b*-siRNA, *inos*-siRNA were infected with *A. hydrophila*. The HKMs were washed, total RNA extracted using TRI reagent (Sigma) from which cDNA was prepared as described earlier [14]. RT-qPCR (ABI ViiA) was done using SYBR green PCR Master Mix (ABI) with specific primers (Table 2). Comparative ∆∆CT method was used to study the expression of target genes and normalized against β-actin used as a housekeeping gene as described earlier [14].

The absolute quantification of cytosolic mtDNA was done by RT-PCR using primers specific for mitochondrial gene *cox2*. Different concentrations of mtDNA (0.1 ng, 0.5 ng, 1 ng, 5 ng, and 10 ng) was used to generate a standard curve. The concentration of cytosolic mtDNA in the samples was interpolated from the standard curve using ct values.

### Protein transfection

DNase 1 (3 μg) or heat-inactivated DNase I (3 μg) was transfected into HKM (1 × 10^7^/mL) using PULSin^TM^ Reagent (Polyplus transfection^TM^) for 4 h at 30°C [27]. HKM viability was checked, infected with *A. hydrophila* and proceeded for subsequent studies.

### Caspase-1 and caspase-3 assay

Caspase-1 activity (YVADase) and caspase-3 activity (DEVDase) were studied using caspase-1 and caspase-3 assay kit (Biovision). HKMs (1 × 10^7^/mL) pre-incubated with indicated inhibitors or transfected with specific siRNA, mtDNA, DNase I, heat-inactivated DNase I, were infected with *A. hydrophila*. HKMs were washed, lysed (at 12 h p.i. for caspase-1 and at 24 h p.i. for caspase-3) and 50 μL of cell lysate was mixed with 2 × reaction buffer containing 5 μL of YVAD-*p*NA substrate (Caspase-1) and DEVD-*p*NA substrate (Caspase-3). The absorbance was recorded at A_405nm_.

### IL-1β assay

HKMs (1 × 10^7^/mL) incubated with inhibitors, or transfected with *il1b*-siRNA or sc-siRNA, mtDNA, DNase I, heat-inactivated DNase I, respectively, were infected with *A. hydrophila*. The changes in IL-1β levels in the culture supernatant were determined using IL-1β assay kit (SunLong Biotech) following the instructions specified by the manufacturer. The IL-1β concentration in the samples was quantified from the standard curve.

### NO production

HKMs (1 × 10^6^/mL) transfected separately with sc-siRNA, *il1b*-siRNA, *inos*-siRNA were infected with *A. hydrophila* and at 24 h p.i., NO production was quantitated using Griess’ reagent [3]. The quantity of nitrite produced was interpolated from sodium nitrite standard curve.

### Apoptosis studies

HKMs (1 × 10^6^/mL) incubated with inhibitors or transfected with specific or sc-siRNA were infected with *A. hydrophila*. HKMs were washed at 24 h p.i. and stained with Hoechst 33,342. The number of apoptotic HKM was calculated under fluorescence microscope (× 40, Zeiss Imager Z2) [14].

### Statistical analysis

The statistical analyses were done using one-way ANOVA followed by Bonferroni post-hoc test (IBM SPSS Software, Version 22). The data are presented as mean ± SE. The *p* value < 0.05 were considered statistically significant.

## Supplementary Material

Supplemental Material

## Data Availability

Data sharing not applicable – no new data generated.
