# Supplementary figures and images for: Aeromonas hydrophila inhibits autophagy triggering cytosolic translocation of mtDNA which activates the pro-apoptotic caspase-1/IL-1β-nitric oxide axis in headkidney macrophages
Source: Virulence. 2021 Dec 30;13(1):60–76. doi: 10.1080/21505594.2021.2018767 (PMC9794009; doi:10.1080/21505594.2021.2018767)

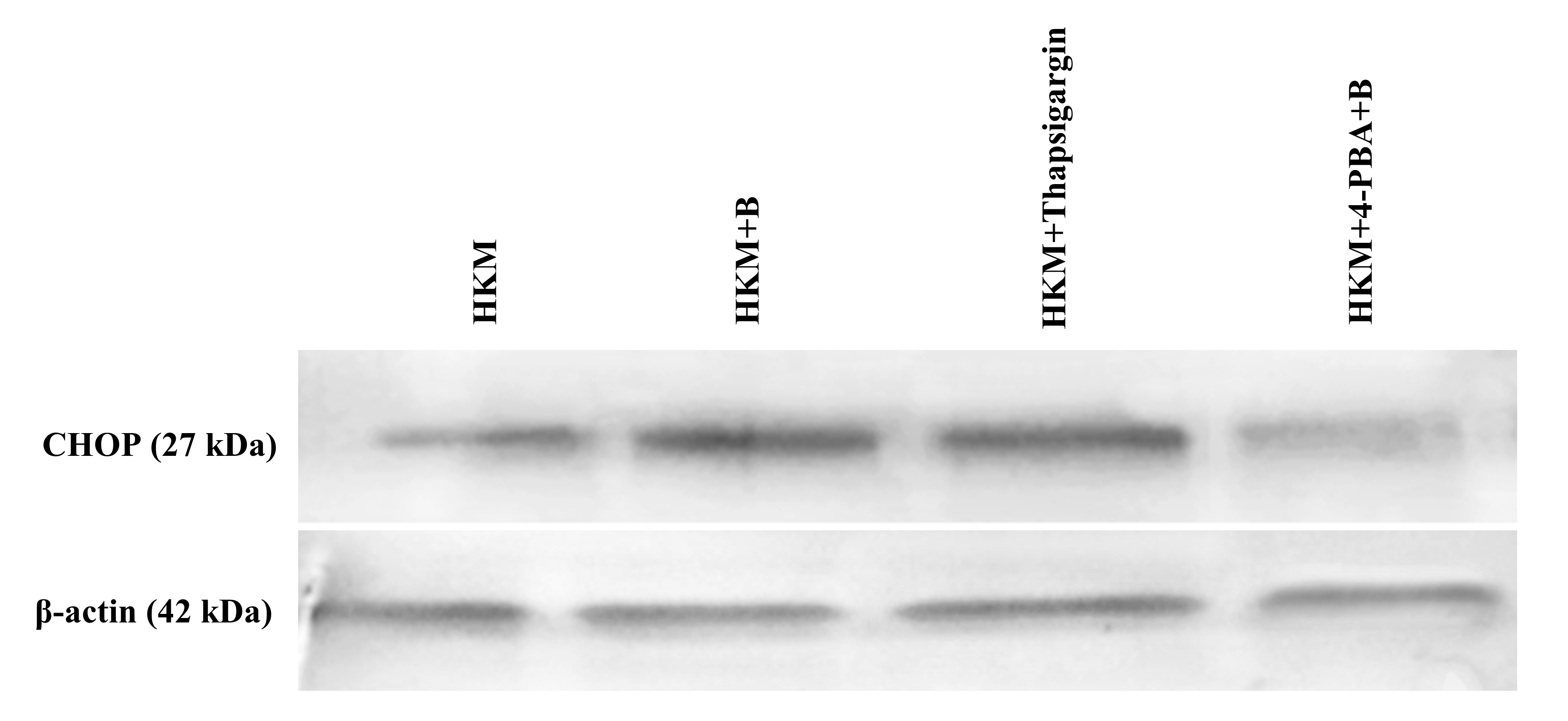

Supplement: Supplemental Material [file KVIR_A_2018767_SM8496.zip › supplementary/Figure S1.tif]

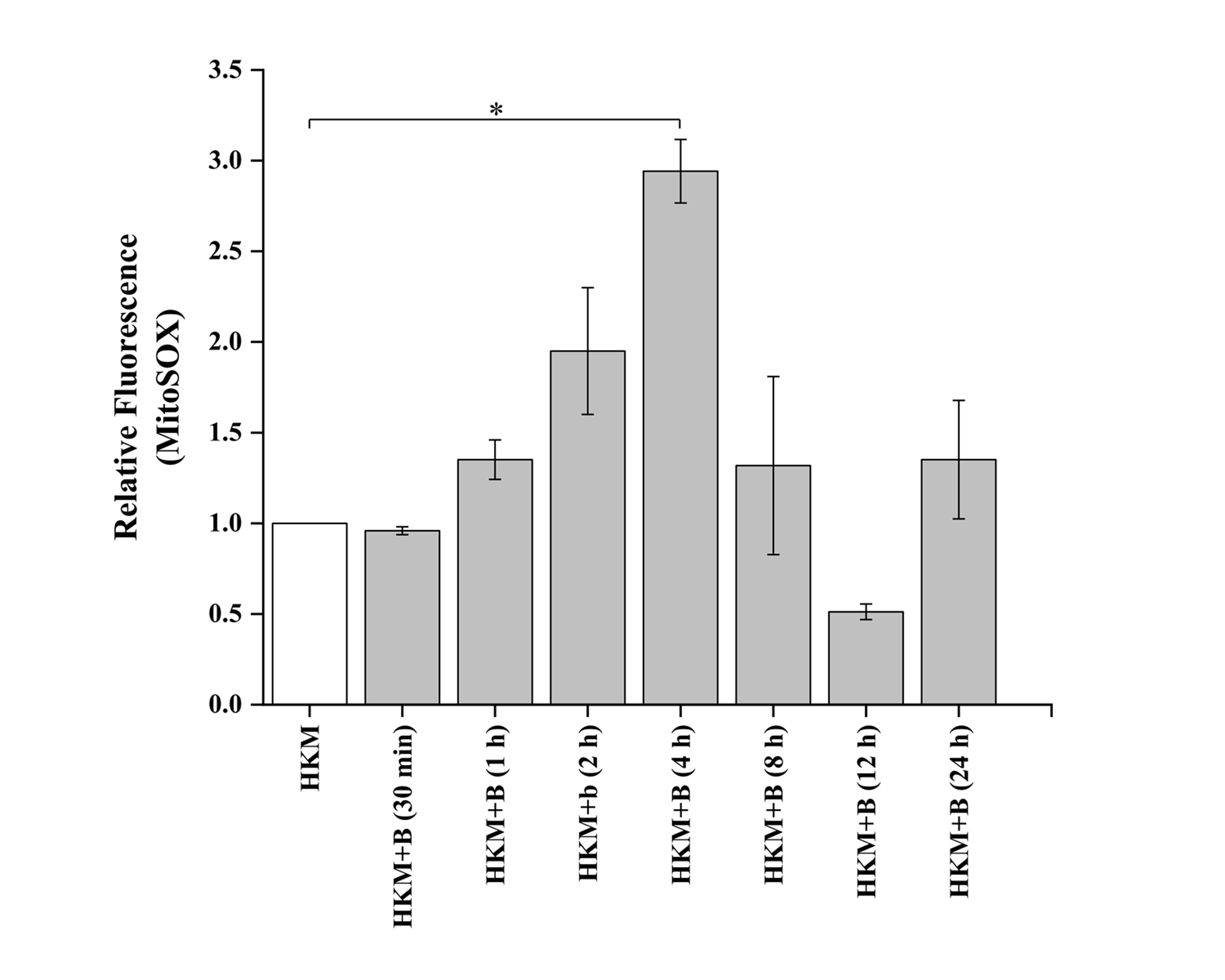

Supplement: Supplemental Material [file KVIR_A_2018767_SM8496.zip › supplementary/Figure S2.tif]
